# Transporters used by parasites as drug targets: tempting but underexploited

**DOI:** 10.1186/1475-2875-9-S2-I9

**Published:** 2010-10-20

**Authors:** Henry Staines

**Affiliations:** 1 Centre for Infection, Division of Clinical Sciences, St George's University of London, Cranmer Terrace, London SW17 0RE, UK

## 

Transport proteins are required to obtain nutrients, remove waste products and regulate cellular volume and ion balances. They make a significant contribution to the relatively small number (< 350) of all known protein drug targets. Of the 186 human targets for FDA-approved oral drugs, 24 (13%) are transporters. However, they remain critically under-researched in the design of antimalarial interventions. As well as being excellent targets in their own right, transporters can provide delivery routes for drugs that target other essential parasite processes and are involved in mediating drug resistance. Therefore, there is ample scientific justification to increase research activity in this area.

For parasite-encoded transporters, well over 100 known or proposed transport protein sequences have been identified in the *P. falciparum* genome (termed the *Plasmodium* "permeome"), with orthologues for the majority appearing in the other plasmodial genomes. Of these, many are considered to have high therapeutic potential such as 11 channels, 13 P-type ATPases and 11 ABC transporters. Yet, only a handful of the proteins within the *Plasmodium* permeome have been characterised in the detail required for assessment of therapeutic targeting. The *P. falciparum* hexose transporter, PfHT, is arguably the best studied parasite transporter. Here are presented recent studies on this potential antimalarial target, utilising the rodent *P. berghei* model to investigate the physiological importance of D-glucose transport in transmission and liver life cycle stages and to generate an *in vivo* model for testing inhibitors of PfHT.

*Plasmodium* parasites may also utilise endogenous host transporters, offering further targeting opportunities. New evidence from liver stage parasites, using high-throughput microarray technology to determine the effect of *P. berghei* infection on the transcriptional profile of host liver cells, has identified over 60 host transport proteins or regulatory subunits that are differentially expressed during the course of infection. These proteins and the first characterisation of transport across the host plasma membrane in *Plasmodium*-infected liver cells will also be discussed.

